# Supplementary material for: First 72-hours after birth: Newborn feeding practices and neonatal mortality in India
Source: PLoS One. 2023 Oct 5;18(10):e0292353. doi: 10.1371/journal.pone.0292353 (PMC10553319; doi:10.1371/journal.pone.0292353)
Supplement: Attachment — Submitted filename: Reviewers comment.docx [file pone.0292353.s001.docx]

**Review of the Paper Titled: First 72-hours after birth: Newborn feeding practices and neonatal mortality in India**

The present paper is of relevance in context of the child health in early years of life. While much of the literature concentrate on analysing the infant and child mortality, the present paper engages with the crucial first 72 hours of life. This also the period which is influenced by the socio-cultural background of the mother. Initiation of breastfeeding as well as the substance to be fed is highly determined by it. The present paper has used robust methodology to examine the feeding practices and neonatal mortality in India evident from the NFHS data of three selected rounds. The analysis bring out the nuances of the early neonatal stage and related mortality.

The following points may be considered for value addition-

**In lines 28-29 as given below🡪**

28 The mortality within the first month of life (during the neonatal period) has shown relatively

29 slow progress

**Observation/Suggestion🡪** *‘Progress in mortality’* is certainly not what the authors intend. Therefore, this line needs to be re-stated as- The mortality **decline** within the first month…slow progress.

**In line 34 as given below🡪**

34 We used data from three rounds of National Family Health Surveys in India.

**Observation/Suggestion🡪** May be a good idea to mention the three rounds of NFHS used in the paper.

**In line 332-334 as given below🡪**

332………	The study further noted, that the pre-lacteal feed did

333 not influence neonatal survival. It is suggested that there is a need to strengthen the intervention of early

334 breastfeeding initiation.

**Observation/Suggestion🡪** It is an important finding that *‘the pre-lacteal feed did not influence neonatal survival’*. Therefore, (a) a couple of lines explanting the phenomenon; and (b) giving viable suggestions for strengthening early breastfeeding will add value to the paper. Similarly for lines 339-340 too, concrete suggestions will be useful for policy makers.

SANGHMITRA S ACHARYA -ORCID ID

[https://orcid.org/0000-0001-6488-4181](https://orcid.org/0000-0001-6488-4181?lang=en)
