# Supplementary material for: First 72-hours after birth: Newborn feeding practices and neonatal mortality in India
Source: PLoS One. 2023 Oct 5;18(10):e0292353. doi: 10.1371/journal.pone.0292353 (PMC10553319; doi:10.1371/journal.pone.0292353)
Supplement: Attachment — Submitted filename: Reviewer comments_PONE-D-22-34236_Abhishek Kumar.docx [file pone.0292353.s002.docx]

**REPORT TEMPLATE**

**Paper title: First 72-hours after birth: Newborn feeding practices and neonatal mortality in India**

**Manuscript no: PONE-D-22-34236**

**Reviewer's report**

The manuscript addressed trends, spatial variation, and determinants of early initiation of breastfeeding and pre-lacteal feeding in India. On very short, the manuscript also analyzed the effect of these breastfeeding practices on neonatal mortality. The study used the three latest rounds of the National Family Health Survey (NFHS) data conducted during 2005-06, 2015-16, and 2019-21. The study applied bivariate and multivariate (the Tobit regression analysis) analysis to examine the level, trends, and determinants of breastfeeding practices.

While reviewing this manuscript, I found that there is a mismatch between the title and the analysis presented in the manuscript. While the title indicates that this manuscript focused on what is the effect of breastfeeding practices on neonatal mortality. However, most of the result section focused on the level, trends, and determinants of the early initiation of breastfeeding and pre-lacteal feeding, and very less is talked about the effect of these on neonatal mortality. This is completely different than what the method section of the abstract claims. Therefore, my suggestion is please remove the neonatal mortality part and only focus on the level, trends, and determinants of the feeding practices. That itself will be a good enough material for a paper. Below is my specific observation on the paper.

**Abstract:**

1. In the method section, please mention the timing of the survey period. Some readers may wonder which three rounds, the first three or the last three out of the five rounds of the National Family Health Survey (NFHS). Though I noticed that it was mentioned in the results section, however, it’s always good to mention the survey date in the method section.
2. Please mention and define the outcome variable in the method section.
3. Please provide some numeric values in the result section.
4. Some parts of the result section can be moved into the discussion or in the conclusion. Such as “While the prevalence of pre-lacteal feed reduced and initiation of early breastfeeding increased considerably after the launch of National Rural Health Mission in India….”.
5. It would be good to present the results and conclusion separately.

**Introduction:**

6. Is the first sentence complete?? “The past few decades have witnessed rapid improvements in the global health scenario as a result of mortality reduction in most populations, including lagers”. Please check. Further, the very first sentence of the introduction section is talking about the reduction in overall mortality. Isn’t it?? If so, then my suggestion is please start talking about childhood mortality or neonatal mortality.
7. Please abbreviate ‘UNIGME’ as many readers may not be aware of it.
8. The “)” at the last of the first paragraph seems unnecessary, please check and remove it.

**Data and method:**

9. In the very first sentence, please write ‘2005-6’ as “2005-06”. Also please provide the survey period of each of the last three rounds of the NFHS.
10. Nothing was mentioned about neonatal mortality. Is neonatal mortality, not your dependent variable? You may consider ‘Early initiation’ as well as ‘Pre-lacteal feed’ as predictors, and independent variables as confounders or independent. Moreover, please provide some reference that why you are considering these independent variables in the analysis.

**Results:**

11. From line number 160-185, nowhere the results (numeric value) are reported. Please report the results in those paragraphs and shorten those.
12. In Table 3, for the reference categories, you have mentioned ‘@’ as well as ‘R’. Please keep only one.

**Discussion:**

13. Too much focus is on the early initiation of breastfeeding and pre-lacteal feeding, and almost nothing is talked about neonatal mortality.

**Conclusions:**

14. The conclusion section is focusing on neonatal mortality only.

**Summary of the comments:**

1. Recommendation (Accept, Minor Revision, Major Revision, Reject)

**--Minor revision**

2. Is the manuscript technically sound, and do the data support the conclusions? (Answer options: Yes, No, Partly)

**--Partly**

3. Has the statistical analysis been performed appropriately and rigorously? (Answer options: Yes, No, I don't know, N/A)

**--Yes**

4. Is the manuscript presented in an intelligible fashion and written in standard English? (Answer options: Yes, No)

**--Yes**

5. Review Comments to the Author

**--No**

6. Would you like your identity revealed to the authors of this submission? (Answer options: Yes, No)
**--Up to you**

7. Do you have any potentially competing interests? If none, type "None." Our policy on competing interests can be found at

**--None**
