# Supplementary material for: First 72-hours after birth: Newborn feeding practices and neonatal mortality in India
Source: PLoS One. 2023 Oct 5;18(10):e0292353. doi: 10.1371/journal.pone.0292353 (PMC10553319; doi:10.1371/journal.pone.0292353)
Supplement: Attachment — Submitted filename: Response to reviewers.docx [file pone.0292353.s003.docx]

**Reviewer 1**

**Review of the Paper Titled: First 72-hours after birth: Newborn feeding practices and neonatal mortality in India**

The present paper is of relevance in context of the child health in early years of life. While much of the literature concentrate on analysing the infant and child by the socio-cultural background of the mother. Initiation of breastfeeding as well as the substance to be fed is highly determined by it. The present paper mortality, the present paper engages with the crucial first 72 hours of life. This also the period which is influenced has used robust methodology to examine the feeding practices and neonatal mortality in India evident from the NFHS data of three selected rounds. The analysis bring out the nuances of the early neonatal stage and related mortality.

**Response 1: We are very grateful to the reviewers for reviewing the manuscript and appreciating the work. We thank reviewer for constructive suggestions and comments. We have incorporated all these valuable suggestions in revised manuscript.**

The following points may be considered for value addition-

**Comment 1:**

**In lines 28-29 as given below🡪**

28 The mortality within the first month of life (during the neonatal period) has shown relatively

29 slow progress

**Observation/Suggestion🡪** *‘**Progress in mortality’* is certainly not what the authors intend. Therefore, this line needs to be re-stated as- The mortality **decline** within the first month…slow progress.

**Response 2: Thanks for pointing this out. We have replaced ‘Progress in mortality’ with ‘mortality decline’. [please see line no. 27].**

**Comment 2:**

**In line 34 as given below🡪**

34 We used data from three rounds of National Family Health Surveys in India.

**Observation/Suggestion🡪** May be a good idea to mention the three rounds of NFHS used in the paper.

**Response 3: Thanks for the suggestion. We have mentioned the last three rounds of NFHS. [please see line nos. 33-34]**

**Comment 3:**

**In line 332-334 as given below🡪**

332………	The study further noted, that the pre-lacteal feed did

333 not influence neonatal survival. It is suggested that there is a need to strengthen the intervention of early

334 breastfeeding initiation.

**Observation/Suggestion🡪** It is an important finding that *‘the pre-lacteal feed did not influence neonatal survival’*. Therefore, (a) a couple of lines explanting the phenomenon; and (b) giving viable suggestions for strengthening early breastfeeding will add value to the paper. Similarly for lines 339-340 too, concrete suggestions will be useful for policy makers.

**Response 4: Thanks for the suggestion. We have added a line in the revised manuscript and have explained the phenomenon [please see line nos. 398-400] and provided viable suggestions for strengthening early breastfeeding component for the future programme [please see line nos. 383-385, 400-402].**

**We have also provided suggestions that may be useful for policy makers [please see line nos. 389-393].**

SANGHMITRA S ACHARYA -ORCID ID

[https://orcid.org/0000-0001-6488-4181](https://orcid.org/0000-0001-6488-4181?lang=en)

**Reviewer 2**

**Paper title: First 72-hours after birth: Newborn feeding practices and neonatal mortality in India**

**Manuscript no: PONE-D-22-34236**

**Reviewer's report**

The manuscript addressed trends, spatial variation, and determinants of early initiation of breastfeeding and pre-lacteal feeding in India. On very short, the manuscript also analyzed the effect of these breastfeeding practices on neonatal mortality. The study used the three latest rounds of the National Family Health Survey (NFHS) data conducted during 2005-06, 2015-16, and 2019-21. The study applied bivariate and multivariate (the Tobit regression analysis) analysis to examine the level, trends, and determinants of breastfeeding practices.

While reviewing this manuscript, I found that there is a mismatch between the title and the analysis presented in the manuscript. While the title indicates that this manuscript focused on what is the effect of breastfeeding practices on neonatal mortality. However, most of the result section focused on the level, trends, and determinants of the early initiation of breastfeeding and pre-lacteal feeding, and very less is talked about the effect of these on neonatal mortality. This is completely different than what the method section of the abstract claims. Therefore, my suggestion is please remove the neonatal mortality part and only focus on the level, trends, and determinants of the feeding practices. That itself will be a good enough material for a paper. Below is my specific observation on the paper.

**Response 5: Thank you for your suggestion and comment. In the revised manuscript we have made necessary changes/modifications. However, the referred table and text, demonstrate that there is a strong association between neonatal mortality and breastfeeding practices as well as pre-lacteal feeding practice. Therefore, we have not removed neonatal mortality part.**

**Abstract:**

**Comment 4:**

In the method section, please mention the timing of the survey period. Some readers may wonder which three rounds, the first three or the last three out of the five rounds of the National Family Health Survey (NFHS). Though I noticed that it was mentioned in the results section, however, it’s always good to mention the survey date in the method section.

**Response 6: Thank you for your suggestion. We have mentioned the survey date in the method section of the abstract. [please see line nos. 33-34]**

**Comment 5:**

Please mention and define the outcome variable in the method section.

**Response 7: Thank you for your suggestion. We have mentioned the outcome variable in the method section of abstract [please see line nos. 35-36]. Detailed definitions of the outcome variables are already mentioned in the method section of the complete manuscript [please see line nos. 106-112].**

**Comment 6:**

Please provide some numeric values in the result section.

**Response 8: Thank you for your suggestion.** **We have included numeric values as necessary in the result section of the abstract. [please see line nos. 38-39]**

**Comment 7:**

Some parts of the result section can be moved into the discussion or in the conclusion. Such as “While the prevalence of pre-lacteal feed reduced and initiation of early breastfeeding increased considerably after the launch of National Rural Health Mission in India….”.

**Response 9: Thank you for your suggestion. We have moved this section into the conclusion. [please see line nos. 46-48]**

**Comment 8:**

It would be good to present the results and conclusion separately.

**Response 10: Thank you for your suggestion. We have moved this part under a new head ‘conclusion’. [please see line no. 45]**

**Introduction:**

**Comment 9:**

Is the first sentence complete?? “The past few decades have witnessed rapid improvements in the global health scenario as a result of mortality reduction in most populations, including lagers”. Please check. Further, the very first sentence of the introduction section is talking about the reduction in overall mortality. Isn’t it?? If so, then my suggestion is please start talking about childhood mortality or neonatal mortality.

**Response 11: Thanks for pointing out. We have modified the sentence [please see line nos. 53-55]. We have replaced ‘mortality’ by ‘childhood mortality’ as suggested. [please see line no. 54]**

**Comment 10:**

Please abbreviate ‘UNIGME’ as many readers may not be aware of it.

**Response 12: Thank you for your suggestion. We have mentioned the full form of ‘UNIGME’. [please see line no. 60]**

**Comment 11:**

The “)” at the last of the first paragraph seems unnecessary, please check and remove it.

**Response 13:** **Thanks for pointing out the typo. We have removed “)”. [please see line no. 62]**

**Data and method:**

**Comment 12:**

In the very first sentence, please write ‘2005-6’ as “2005-06”. Also please provide the survey period of each of the last three rounds of the NFHS.

**Response 14: Thank you for your suggestion. We have ensured consistency throughout the manuscript and corrected the year as ‘2005-06’. [please see line nos. 94 and 216]. We have provided survey periods. [please see line nos. 93-94 and response 6]**

**Comment 13:**

Nothing was mentioned about neonatal mortality. Is neonatal mortality, not your dependent variable? You may consider ‘Early initiation’ as well as ‘Pre-lacteal feed’ as predictors, and independent variables as confounders or independent. Moreover, please provide some reference that why you are considering these independent variables in the analysis.

**Response 15: Thank you for your suggestion. We have added a few lines about neonatal mortality in the method section [please see line nos. 137-145] and also included two references by Edmond et al (2008) and Phukan et al (2018). [please see line nos. 137-140]**

**Results:**

**Comment 14:**

From line number 160-185, nowhere the results (numeric value) are reported. Please report the results in those paragraphs and shorten those.

**Response 16: Thank you for your suggestion. We have included numeric values. [please see line nos. 186-188, 193-195, 200, 202-203, 206-207]**

**Comment 15:**

In Table 3, for the reference categories, you have mentioned ‘@’ as well as ‘R’. Please keep only one.

**Response 17: Thank you for your suggestion. We have now used ® to indicate reference category in the Table 3.**

**Discussion:**

**Comment 16:**

Too much focus is on the early initiation of breastfeeding and pre-lacteal feeding, and almost nothing is talked about neonatal mortality.

**Response 18: Thank you for your suggestion. We have added a paragraph about neonatal mortality. [please see line nos. 365-368, 370-376]**

**Conclusions:**

**Comment 17:**

The conclusion section is focusing on neonatal mortality only.

**Response 19: Thank you for your suggestion. We have now modified conclusion and included breastfeeding practices as well as pre-lacteal feed. [please see line nos. 386-393, 398-400].**
