# Supplementary material for: First 72-hours after birth: Newborn feeding practices and neonatal mortality in India
Source: PLoS One. 2023 Oct 5;18(10):e0292353. doi: 10.1371/journal.pone.0292353 (PMC10553319; doi:10.1371/journal.pone.0292353)
Supplement: Attachment — Submitted filename: Manuscript_revised_edited.docx [file pone.0292353.s004.docx]

**First 72-hours after birth: Newborn feeding practices and neonatal mortality in India**

Piyasa Mal^1^, Usha Ram^2*^

^1^ Department of Public Health and Mortality Studies, International Institute for Population Sciences, Mumbai, Maharashtra, India

^2^ Department of Biostatistics and Epidemiology, International Institute for Population Sciences, Mumbai, Maharashtra, India

* Corresponding author

Email: usharamagrawal@gmail.com

The authors have declared that no competing interests exist.

Author's contribution: UR conceptualized the paper. PM conducted the analysis under the supervision and guidance of UR. UR and PM developed the draft manuscript. UR finalized the manuscript for submission.

The authors received no specific funding for this work.

**Abstract**

## **Background**

The reductions in mortality levels among children under five years are observed in most populations, including populations that were lagging the progress in the past. However, the reduction is not uniform across ages during childhood. The mortality declines within the first month have shown relatively slow progress. Early initiation of breastfeeding and discarding pre-lacteal feed protects the newborn from acquiring infection and, thereby, reduces mortality. This paper assesses the change in the prevalence of early initiation of breastfeeding and pre-lacteal feed along with their associated factors, and associated neonatal mortality in India.

## **Methods**

We used data from the last three rounds of National Family Health Surveys conducted during 2005-06, 2015-16 and 2019-21 in India. We used bivariate and multivariate analyses to examine prevalence rates, risk factors, and relationships between breastfeeding practices including early initiation of breastfeeding and pre-lacteal feed, and neonatal mortality.

## **Results**

Early initiation of breastfeeding within one hour after birth increased rapidly from 25% in 2005-06 to 42% in 2019-21 and the pre-lacteal feeding practice declined from 57% in 2005-06 to 15% in 2019-21. Pre-lacteal feed is lower in states/districts where early breastfeeding initiation is predominant and vice versa. The role of health professionals during pregnancy and the first two days after delivery significantly improved breastfeeding practice. Further, the findings suggest that an early breastfeeding initiation is associated with lower neonatal mortality, whereas pre-lacteal feed is not harmful as compared to late breastfeeding initiation.

**Conclusion**

Prevalence of pre-lacteal feed reduced, and initiation of early breastfeeding increased considerably after the launch of the National Rural Health Mission in India. However, after 2015-16, early breastfeeding initiation has stagnated, and the decline in pre-lacteal feed has slowed down. The future program needs special attention to emphasize the availability and accessibility of breastfeeding advisers and observers in health facilities to help mitigate adverse neonatal outcomes.

# **Introduction**

The past few decades have witnessed rapid improvements in the global health scenario as a result of childhood mortality reduction in most populations. Several countries, including India, for example, have made remarkable progress in mortality reductions among children below five years of age (under-five mortality rate, U5MR), especially during the past decade. The global child mortality rate has dropped by 60% between 1990 and 2020, from 93 to 37 per 1,000 live births [1]. However, the available evidence suggests that the reduction is slower in mortality within the first month of life. The United Nations Inter-agency Group for Child Mortality Estimation-UNIGME (2021) estimates indicate that globally the neonatal mortality rate (NMR) has dropped from 37 per 1,000 live births in 1990 to 17 per 1,000 live births in 2020, yet contributed 47% of all under-five deaths [2].

In 2020, India accounted for the highest number of global neonatal deaths (490 thousand; 20% of global neonatal deaths) [3]. In India, neonatal deaths comprised almost half of all under-5 deaths in 2000, which rose 62% in 2020 [4]. Globally as well as in India, prematurity and low birth weight, neonatal infections (neonatal pneumonia, neonatal sepsis, and central nervous system infections), and birth asphyxia and birth trauma account for more than three-quarters of all neonatal deaths [5].

Early initiation of breastfeeding protects the newborn from acquiring infections and reduces mortality [6–8]. It further strengthens the emotional bonding between the newborn and mother and positively impacts the duration of exclusive breastfeeding [9,10]. The yellow or golden first milk produced in the first few days (also called colostrum) is an essential source of nutrition and immune protection for the newborn. Colostrum is a complex biological nutrient-rich fluid produced by female mammals immediately after giving birth that provides immune, growth, and tissue repair capabilities [11,12]. Early breastfeeding initiation can significantly contribute to the achievement of the child survival [13].

Breastfeeding among Indian mothers is nearly universal; however, myths and superstitions such as discarding colostrum, delayed initiation of breastfeeding, pre-lacteal feeding, and early initiation of complementary feeding persist [14,15]. Several social, cultural, and economic factors, including maternal education and employment, maternal age, attitude and conﬁdence, prenatal intention, ethnicity, residence, type of family, emulating western lifestyles, the inﬂuence of healthcare professionals, and availability of infant formula, influence breastfeeding practices [14,16]. Further, breastfeeding attitudes and practices differ in different segments of society depending on traditional cultural practices and taboos prevalent in them [13]. Timely initiation of breastfeeding remains low in India, and the pre-lacteal feeding is still prevalent [17]. As also reflected from the global estimate (WHO 2021), 3 in 5 babies in India are not breastfed in the first hour of life [18].

India is committed to achieving the targeted reduction in child mortality under the third Sustainable Development Goal (SDG) and making multi-pronged efforts in achieving them. This study assesses breastfeeding practices regarding early initiation and pre-lacteal feeding in India and their association with neonatal mortality. The study specifically appraises i) prevalence of early initiation of breastfeeding and pre-lacteal feeding practices by socio-demographic characteristics of mother and child, and household; ii) predictors of early initiation of breastfeeding and pre-lacteal feeding practices; and iii) how the level of neonatal mortality differs across the status of pre-lacteal feeding and delayed breastfeeding initiation.

# **Methods**

**Data**

We have used data from the last three rounds of the National Family Health Survey (NFHS-3 conducted during 2005-06, NFHS-4 conducted during 2015-16, and NFHS-5 conducted during 2019-21) to analyse trends in the early initiation of breastfeeding and pre-lacteal feed. The details of the survey are available here (<https://dhsprogram.com/data/available-datasets.cfm>). The NFHS-3 (2005-06) surveyed 109,041 households and 124,385 women of reproductive age. From NFHS-4 (2015-16), the survey was implemented at the district levels and interviewed 572,000 households and 699,686 women of reproductive age. The NFHS-5 (2019-21) surveyed 636,699 households and 724,115 women of reproductive age. Data were collected following all the ethical guidelines. During the data collection, ethical approval was granted from the Institutional Review Board (IRB) of the International Institute for Population Sciences (IIPS) and ICF Institutional Review Board. During the field survey, verbal and written consent were taken from the respondents.

**Outcome variables**

The study has two main outcome variables: early initiation of breastfeeding and pre-lacteal feed. The NFHS asked women who had a livebirth during the past five years preceding the survey– ‘how long after birth did you start breastfeeding?’ We considered the ‘early initiation of breastfeeding’ practice if mothers breastfed the child within one hour after birth. Similarly, the survey also asked mothers – ‘In the first three days after delivery, was the baby given anything to drink other than breast milk?’ If mothers reported that they gave child anything other than breast milk, we considered it as ‘pre-lacteal feed’. In addition, neonatal mortality rate was estimated stratified by the status of breastfeeding initiation and pre-lacteal feeding. The study sample constitutes the last-born children of the women of the reproductive age during the last five years preceding the respective surveys.

**Predictor variables**

These predictor variables include: place of residence (rural, urban); sex of the child (male, female); maternal age at birth (≤19, 20-34, ≥35); birth order (1, 2, 3 or more), maternal education (no education, primary, secondary or higher); household wealth tertile (poor, middle, rich); maternal caste/tribe (Scheduled Castes (SCs), Scheduled Tribes (STs), Other Backward Class (OBC), others); mother’s ANC visits during pregnancy (No visit, 1-3, 4 or more); place of delivery (not in a health facility, public health facility, private health facility); types of delivery (vaginal deliveries, C-section in a public facility, C-section in a private facility); mother received breastfeeding advice during pregnancy (no, yes), and healthcare provider observed breastfeeding during first two days after birth (no, yes).

**Statistical analysis**

By applying appropriate sampling weights, we conducted bivariate analyses to assess the levels of early initiation of breastfeeding and pre-lacteal feeding practice by socioeconomic and demographic characteristics of the mother, child, and household. We used Chi-squared tests to assess statistical significance of bivariate associations of outcome variables with the socioeconomic and demographic characteristics.

As NFHS provides information on pre-lacteal feeding and initiation of breastfeeding only for the youngest child, we created two groups, one consisting of children of last birth order about whom we had information on both regressors (predictor variables) and regressand (outcome variables). Second group consisted of children of previous birth order about whom we only had information on the regressors but not on the regressand. We applied Tobit regression model to examine the probability of early initiation of breastfeeding and pre-lacteal feeding practice concerning exposure variables using the pooled datasets of the last three rounds of the NFHS covering the past two decades.

Statistically, we can express the Tobit model as

$YiY_{i}=\beta_{1}+ \beta_{2}X_{i} + \beta_{3}X_{i}\ldots\ldots+ui$ if RHS (right-hand side) > 0

= 0	otherwise

We tested all exposure variables for possible multi-collinearity before inclusion into the Tobit regression model.

Two studies, viz. Edmond et al. [19] and Phukan et al. [20] have examined impact of breastfeeding practices on child mortality. While Edmond et al. [19] found that the pre-lacteal feeding increased infant mortality in rural Ghana, Phukan et al. [20] observed that the neonatal mortality was higher among neonates with delayed initiation of breastfeeding in India. In view of the paucity of studies, the present study conducted a bivariate analysis to examine association between levels of neonatal mortality by timing of initiation of breastfeeding and pre-lacteal feeding practices in Indian population using the data for the period 2019-21.

We conducted the analyses using STATA version 16 [21]. Maps were created using ArcMap 10 [22]. The shapefiles of the maps were accessed from the official website of Demographic Health Survey (<https://dhsprogram.com/data/available-datasets.cfm>).

# **Results**

## **Levels and trends in early initiation of breastfeeding and pre-lacteal feeding: National, sub-national and by population subgroups**

Early initiation of breastfeeding within one hour after birth increased rapidly from just about one-quarter in 2005-06 to nearly 42% in 2019-21 and the percentage of newborns given a pre-lacteal feed declined from more than 57% in 2005-06 to 15% in 2019-21 (Figure 1).

**Figure 1: Early initiation of breastfeeding and pre-lacteal feed in India, 2005-06 to 2019-21**

In sixteen of the 30 Indian states in 2005-06, fewer than 35% of mothers fed breastmilk to their newborns immediately after birth (Figure 2). However, in 11 states (Maharashtra, Kerala, Tamil Nādu, Orissa, and the north-eastern states), more than 50% of the mothers breastfed their newborns immediately after birth. The early initiation of breastfeeding improved dramatically over time. By 2019-21, more than half of the mothers initiated early breastfeeding in 37% of districts, and in another 28% of districts 35-50% of mothers did so. Nonetheless, fewer than 35% of newborns were breastfed immediately after birth in about 35% of districts, mostly in Uttar Pradesh, Bihar, Jharkhand, Rajasthan, and Gujarat.

**Figure 2: Early initiation of breastfeeding (within 1 hour after birth) and pre-lacteal feed after birth** **across states/districts in India, 2005-06 to 2019-21**

Pre-lacteal feed shows a downward trend in all the states/districts of India. In 2005-06, more than 25% of newborns were given pre-lacteal feed in 23 states, including Uttar Pradesh, Bihar, Punjab, Rajasthan, Madhya Pradesh, West Bengal etc. By 2019-21, the proportions reduced tremendously. For example, more than 25% newborns received pre-lacteal feed in about 11% of the Indian districts (mostly concentrated in Uttar Pradesh). In two states (Kerala and Sikkim) in 2005-06, fewer than 12.5% of newborns were given a pre-lacteal feed. In nearly 46% of the districts in 2019-21, fewer than 12.5% of newborns were given a pre-lacteal feed.

Compared to the children born to mothers in rural areas, children in urban areas had an advantage, as higher proportion of urban mothers initiated early breastfeeding and fewer gave a pre-lacteal feed (Table 1). For example, 44% of urban mothers initiated breastfeeding within one hours compared to 41% of rural mothers in 2019-21. Similarly, 15% of urban mothers gave a pre-lacteal feed to newborn compared 17% in rural areas. The feeding practice did not differ by newborn’ gender. While early initiation of breastfeeding was higher and, pre-lacteal feed was lower among children born to younger mothers and children of lower birth order (1 or 2). Similarly, children born to mothers with secondary or highest education, belonged to rich wealth tertile had huge advantage over those born to mothers with no education or born to mothers in poor wealth tertile as more received early breastmilk and fewer were given a pre-lacteal feed. For example, in 2019-21, 37% children born to mothers who did not receive any education and 43% children born to secondary or higher received early breastmilk. The corresponding numbers for children who received a pre-lacteal feeding is 15% and 16%, respectively. Further, children born to scheduled tribe mothers received early breastmilk and fewer received a pre-lacteal feed than the children born to mothers of other caste groups.

In 2019-21, the early initiation of breastfeeding was higher among children whose mothers received 4 or more ANC visits (47%) than those with fewer ANC visits (35%) or no ANC visit (32%). Conversely, fewer children (15%) were given a pre-lacteal feed if the mothers received 4 or more ANC visits during pregnancy compared to those who received fewer or no ANC visit (17% and 15%, respectively) in 2019-21. Relatively higher proportions of mothers initiated early breastfeeding and fewer gave a pre-lacteal feed if the delivery occurred in a public health facility. In contrast, only fewer children were breastfed early and more received a pre-lacteal feed if the delivery did not occur in a health facility. Similarly, higher proportions of children were early breastfed (44%) and fewer were given a pre-lacteal feed (12%) if the delivery was vaginal.

The percentage of children who were breastfed early was higher among mothers who were advised about early initiation of breastfeeding during the pregnancy. At the same time, fewer mothers who received advice gave pre-lacteal feed to the newborn. Similarly, feeding practices were better when a healthcare provider observed breastfeeding during the first 2 days after birth.

Not only have the feeding practices have improved in all population subgroups during the analysis period, the gap in feeding practices across population subgroups too have narrowed. For example, the relative disadvantage in rural children has reduced from 8% points in 2005-06 to 3% points in 2019-21. Similarly, relative disadvantage of pre-lacteal feed reduced from about 10% points to 3% points during the same period.

Table 1. Percentage of children* who breastfed within one hour after birth and who received pre-lacteal feeding by selected socio-demographic characteristics, India, 2005-06 to 2019-21

|  | Early initiation of breastfeeding | | | Pre-lacteal feeding | | |
| --- | --- | --- | --- | --- | --- | --- |
|  | 2005-06 | 2015-16 | 2019-21 | 2005-06 | 2015-16 | 2019-21 |
| **Overall** | **24.5** | **41.4** | **41.7** | **57.2** | **20.8** | **15.3** |
| Place of residence | |  |  |  |  |  |
| Rural | 22.4 | 40.9 | 40.7 | 57.6 | 20.6 | 17.1 |
| Urban | 30.3 | 42.6 | 44.2 | 48.0 | 21.2 | 14.6 |
| Sex of child | |  |  |  |  |  |
| Male | 24.7 | 41.0 | 41.7 | 55.2 | 21.0 | 15.6 |
| Female | 24.3 | 41.9 | 41.7 | 54.9 | 20.5 | 14.9 |
| Maternal age at birth (in years) | | |  |  |  |  |
| ≤19 | 22.3 | 42.6 | 45.7 | 56.9 | 18.7 | 14.0 |
| 20-34 | 25.3 | 41.6 | 41.6 | 53.9 | 20.7 | 15.3 |
| ≥35+ | 18.3 | 36.6 | 38.3 | 66.5 | 25.6 | 16.0 |
| Birth order | |  |  |  |  |  |
| 1 | 26.5 | 41.2 | 41.1 | 52.0 | 22.0 | 18.0 |
| 2 | 30.0 | 43.9 | 43.8 | 47.2 | 18.0 | 14.0 |
| 3 or higher | 19.8 | 39.0 | 39.9 | 61.9 | 22.5 | 13.7 |
| Maternal education | |  |  |  |  |  |
| No education | 16.7 | 35.9 | 36.8 | 65.0 | 23.8 | 14.7 |
| Primary | 26.1 | 40.6 | 41.9 | 51.7 | 20.4 | 13.5 |
| Secondary or higher | 33.4 | 44.2 | 43.1 | 44.1 | 19.5 | 15.8 |
| Household wealth tertile | | |  |  |  |  |
| Poor | 18.5 | 38.9 | 39.3 | 63.4 | 21.4 | 12.4 |
| Middle | 23.9 | 42.9 | 42.1 | 55.9 | 18.9 | 14.6 |
| Rich | 30.4 | 42.3 | 43.4 | 46.8 | 22.0 | 18.4 |
| Maternal caste/tribe |  |  |  |  |  |  |
| Scheduled castes | 23.2 | 41.2 | 41.9 | 56.7 | 19.7 | 14.2 |
| Scheduled tribe | 28.6 | 45.4 | 46.1 | 42.0 | 12.4 | 10.2 |
| Other backward class | 22.0 | 40.1 | 38.8 | 60.4 | 23.4 | 16.1 |
| Other castes | 26.8 | 41.2 | 43.6 | 51.7 | 22.2 | 18.8 |
| Mother received ANC visits during pregnancy | | |  |  |  |  |
| No visit | 11.3 | 34.0 | 32.4 | 75.3 | 23.8 | 15.4 |
| 1 to 3 visits | 21.1 | 37.2 | 34.9 | 58.1 | 23.3 | 16.5 |
| 4 or more visits | 35.8 | 46.4 | 46.5 | 40.0 | 18.4 | 14.7 |
| Place of delivery | |  |  |  |  |  |
| Not in a health facility | 17.8 | 34.3 | 34.2 | 64.9 | 29.0 | 20.9 |
| Public health facility | 37.3 | 45.8 | 44.6 | 34.8 | 14.4 | 10.5 |
| Private health facility | 31.1 | 38.1 | 38.1 | 46.6 | 27.2 | 24.4 |
| Type of delivery | |  |  |  |  |  |
| Vaginal deliveries | 24.8 | 43.4 | 43.8 | 55.3 | 18.8 | 12.1 |
| C-section public facility | 25.9 | 37.7 | 39.5 | 43.3 | 19.4 | 17.2 |
| C-section private facility | 20.1 | 30.9 | 32.1 | 56.5 | 34.7 | 31.2 |
| Mother received breastfeeding advice during pregnancy | | | | |  |  |
| No | 19.0 | 34.4 | 37.8 | 61.2 | 28.4 | 19.4 |
| Yes | 36.1 | 47.0 | 44.4 | 40.4 | 16.6 | 13.8 |
| Healthcare providers observed breastfeeding during first 2 days after birth | | | | | |  |
| No | NA | NA | 36.3 | NA | NA | 18.4 |
| Yes | NA | NA | 43.4 | NA | NA | 14.6 |
| N | 38528 | 184641 | 174947 | 38528 | 174,468 | 165,131 |

Notes:

1. The results of χ2 suggest that all variables are significant at less than 1% for both early initiations of breastfeeding and pre-lacteal feeding for all three time periods included in the analysis.
2. Sex of the child is significant at <10% for early initiations of breastfeeding in 2019-21 and is insignificant for the years 2005-06 for both early initiations of breastfeeding and pre-lacteal feeding
3. NA = Not available
4. *last-born child among children born during the past five years preceding the survey

### **Pre-lacteal feed by delivery type**

Regardless of the time period, other milk (not breastmilk) followed by plain water, honey and Sugar, glucose or salt water solution was the most common pre-lacteal feed (Table 2). About 59% of mothers in 2005-06 fed their newborns with other milk (not breastmilk), which increased to 72% in 2019-21. About 11% and 9% of newborns in 2019-21 were fed with plain water and honey, respectively. Another about 8% of mothers in 2019-21 gave sugar, glucose or saltwater solution to the newborns. A minority of mothers also reported that they fed their newborn with *janam ghutti*, tea/infusions, infant formula or gripe water. The choice of pre-lacteal feed varied by delivery type. For example, in 2019-21 higher percentages of mother who had a C-section delivery gave other milk to the newborn than those whose delivery was vaginal (77% versus 69%). Likewise, higher proportion of mothers who had a C-section reportedly gave plain water or honey to the newborn.

Table 2. Distribution of children by types of pre-lacteal feed and delivery type, India, 2005-06 to 2019-21

| Pre-lacteal type | 2005-06 | | | 2015-16 | | | 2019-21 | | |
| --- | --- | --- | --- | --- | --- | --- | --- | --- | --- |
|  | Vaginal | C-section | Overall | Vaginal | C-section | Overall | Vaginal | C-section | Overall |
| Other milk (not breastmilk) | 59.1 | 54.4 | 58.7 | 61.7 | 69.6 | 63.8 | 68.6 | 77.1 | 71.9 |
| Plain water | 15.8 | 15.8 | 15.8 | 12.6 | 11.9 | 12.4 | 12.7 | 8.7 | 11.2 |
| Honey | 25.5 | 16.8 | 24.7 | 15.2 | 9.0 | 13.6 | 10.5 | 6.4 | 8.9 |
| Sugar/glucose/salt water or solution | 26.1 | 30.3 | 23.2 | 11.8 | 12.9 | 12.1 | 8.5 | 7.2 | 8.0 |
| *Janam ghutti* | 8.9 | 3.9 | 8.4 | 8.4 | 4.6 | 7.4 | 7.0 | 3.2 | 5.5 |
| Tea/infusions | 5.6 | 2.6 | 5.3 | 7.2 | 2.1 | 5.8 | 7.4 | 2.3 | 5.4 |
| Infant formula | 0.6 | 7.1 | 1.2 | 2.0 | 6.6 | 3.2 | 2.6 | 7.6 | 4.6 |
| Fruit juice | 0.2 | 0.2 | 0.2 | 0.8 | 0.7 | 0.8 | 1.9 | 1.2 | 1.6 |
| Gripe water | 0.5 | 1.1 | 0.6 | 1.4 | 1.9 | 1.5 | 1.5 | 1.6 | 1.5 |
| Others | 3.8 | 1.8 | 3.6 | 4.0 | 2.6 | 3.6 | 3.2 | 3.3 | 3.3 |

## **Determinants of early initiation of breastfeeding**

Results from Tobit analysis adjusted for other socioeconomic, demographic, and programmatic variables suggest that newborns of mothers aged 20-34 years are less likely to be breastfed within one hour after birth than those born to younger mothers (Table 3). Compared to first born, children of subsequent birth orders are more likely to be breastfed within one hour after birth. Mother’s educational attainment and household economic status had a statistically significant positive association with early initiation of breastfeeding. For example, compared to newborn of uneducated mothers, newborn whose mothers had primary or secondary or higher education were 9% and 13% more likely to have received early breastmilk, respectively. Children born to mothers in middle and rich wealth tertile were 2-3% more likely to have received early breastmilk compared to those born in poor wealth tertile. Compared to the scheduled tribes, newborn from other castes were 16-21% less likely to have received early breastmilk.

Newborn of mothers who had 4 or more ANC visits were 23% more likely to receive early breastmilk than those born to mothers who did not get any ANC visit. Similarly, newborn delivered in a public health facility were 14% more likely to have received early breastmilk than those delivered in a non-health facility. Mothers who had a C-section delivery in a public facility or a private health facility were 25% and 34%, respectively, less likely to early initiate breastfeeding than those who had a vaginal delivery. Mothers who received breastfeeding advice during pregnancy were 19% more likely to early initiate breastfeeding. Early initiation of breastfeeding was more likely among children born in recent times compared to those born earlier (2005-06).

**Determinants of pre-lacteal feed**

Results adjusted for other socioeconomic, demographic, and programmatic variables suggest that newborns of mothers in urban areas were 4% less likely to be given a pre-lacteal feed than those born in rural areas (Table 3). Newborn boys were 3% more likely to receive a pre-lacteal feed than the newborn girls. Newborns to mothers older than 35 years were 14% more likely to get a pre-lacteal feed than those born to younger mothers. Compared to first born, children of subsequent birth order were 14-23% less likely to get a pre-lacteal feed. Compared to newborns of uneducated mothers, newborn whose mothers had primary or secondary or higher education were 9% and 17% less likely to get a pre-lacteal feed, respectively. Newborns to mothers belonging to middle and rich wealth tertile were 10-23% more likely to get a pre-lacteal feed compared to those born in poor wealth tertile. Compared to the Scheduled tribes, the newborns from other castes were 30-36% more likely to get a pre-lacteal feed.

While newborns of mothers who had 4 or more ANC visits were 15% less likely to get a pre-lacteal feed, those born to mothers who got 1-3 ANC visits were 7% more likely to get a pre-lacteal feed than those born to mothers who did not get any ANC visit. Further, newborn delivered in a public health facility were 55% and those born in a private health facility were 9% less likely to get a pre-lacteal feed than those delivered in a non-health facility. Mothers who had a C-section delivery in a public or a private health facility were 53-55% more likely to give a pre-lacteal feed than those who had a vaginal delivery. Mothers who received breastfeeding advice during pregnancy were 41% less likely to give a pre-lacteal feed than those who did not. Compared to those born during 2005-06, likelihood of a pre-lacteal feed was 67% lower among children born during 2015-16 and 85% lower among children born 2019-21.

Table 3. Results of Tobit model showing determinants of early initiation of breastfeeding and pre-lacteal feed, India [Based on Pooled NFHS Data, 2005-06, 2015-16, 2019-21]

|  | Coefficient (95% confidence interval) | |
| --- | --- | --- |
|  | Initiation of breastfeeding | Pre-lacteal feed |
| Place of residence |  |  |
| Rural ® |  |  |
| Urban | 0.01[-0.00,0.02] | -0.04***[-0.06,-0.02] |
| Sex of child |  |  |
| Female ® |  |  |
| Male | -0.01[-0.02,0.00] | 0.03*[0.02,0.05] |
| Maternal age at birth (in years) |  |  |
| ≤19 ® |  |  |
| 20-34 | -0.02*[-0.04,-0.00] | 0.03[-0.00,0.07] |
| ≥35 | 0.02[-0.01,0.04] | 0.14***[0.09,0.19] |
| Birth order |  |  |
| 1 ® |  |  |
| 2 | 0.07***[0.06,0.08] | -0.23***[-0.25,-0.21] |
| 3 or higher | 0.05***[0.04,0.07] | -0.14***[-0.17,-0.12] |
| Maternal education | |  |
| No education ® |  |  |
| Primary | 0.09***[0.07,0.11] | -0.09***[-0.12,-0.06] |
| Secondary or higher | 0.13***[0.12,0.15] | -0.17***[-0.20,-0.15] |
| Household wealth tertile |  |  |
| Poor ® |  |  |
| Middle | 0.03***[0.02,0.04] | 0.10***[0.08,0.13] |
| Rich | 0.02**[0.01,0.04] | 0.23***[0.20,0.25] |
| Maternal caste/tribe |  |  |
| Scheduled Tribe ® |  |  |
| Scheduled Castes | -0.16***[-0.18,-0.15] | 0.30***[0.27,0.33] |
| Other backward class | -0.21***[-0.22,-0.19] | 0.32***[0.29,0.34] |
| Other castes | -0.17***[-0.18,-0.15] | 0.36***[0.33,0.40] |
| Mother received ANC visits during pregnancy | |  |
| No visit ® |  |  |
| 1 to 3 visits | 0.02[-0.01,0.04] | 0.07***[0.03,0.11] |
| 4 or more visits | 0.23***[0.21,0.26] | -0.15***[-0.19,-0.11] |
| Place of delivery |  |  |
| Not in a health facility ® |  |  |
| Public health facility | 0.14***[0.13,0.16] | -0.55***[-0.58,-0.52] |
| Private health facility | 0.02*[0.00,0.04] | -0.09***[-0.13,-0.06] |
| Type of delivery |  |  |
| Vaginal deliveries ® |  |  |
| C- section in a public facility | -0.25***[-0.26,-0.23] | 0.53***[0.50,0.56] |
| C- section in a private facility | -0.34***[-0.36,-0.32] | 0.55***[0.52,0.59] |
| Mother received breastfeeding advice during pregnancy | |  |
| No ® |  |  |
| Yes | 0.19***[0.17,0.20] | -0.41***[-0.44,-0.39] |
| Survey period |  |  |
| 2005-06 ® |  |  |
| 2015-16 | 0.25***[0.22,0.27] | -0.67***[-0.70,-0.63] |
| 2019-21 | 0.18***[0.15,0.20] | -0.85***[-0.89,-0.82] |

® = Reference category, * p<0.05, ** p<0.01, *** p<0.001

## **Neonatal mortality and feeding practices**

The overall estimated neonatal mortality rate (NMR) was 24.8 per 1000 livebirths in India in 2019-21 (Table 4). The NMR among children who were ever breastfed was 6.9 per 1000 livebirths as against of 198 per 1000 livebirths among those who were never breastfed. The NMR was 28.4 per 1000 livebirths among children born to mothers who did not answer on breastfeeding status. The NMR was 6.6 and 6.8 per thousand livebirths among children who were breastfed within one hour after birth or between one hour to one day after birth, respectively. In contrast, the NMR was 75.2 per 1000 livebirths among children who received delayed breastfeeding one day after birth. The NMR was 5.1 per thousand livebirths among newborn who received pre-lacteal feed and 7.1 per 1000 live births among those who did not receive a pre-lacteal feed.

Table 4: Neonatal mortality rate per thousand live births by the breastfeeding and pre-lacteal feeding status, India (2019-21)

| Practices | Neonatal mortality rate (NMR) | Number of live births |
| --- | --- | --- |
| Ever breastfed |  |  |
| Yes | 6.9 | 1,67,606 |
| Never | 197.7 | 16,377 |
| Did not answer | 28.4 | 46,887 |
| Timing of breastfeeding initiation |  |  |
| Within 1 hour | 6.6 | 72,960 |
| 1 hour to 1 day | 6.8 | 77,001 |
| More than 1 day | 75.2 | 24,986 |
| Pre-lacteal feeding (for last birth) |  |  |
| Yes | 5.1 | 25,212 |
| No | 7.1 | 1,39,919 |
| Did not answer | 178.9 | 9,816 |
| Overall | 24.8 | 2,30,870 |

# **Discussion**

The study documents a spatial pattern in initiation of early breastfeeding and pre-lacteal feed in India. The pre-lacteal feeding practice is less common in regions where early initiation of breastfeeding is predominant and vice versa. The early breastfeeding initiation is more commonly prevalent in southern India where pre-lacteal is less prevalent. In contrast, initiation of early breastfeeding is lower and pre-lacteal is higher in the northern India. Mothers who practiced pre-lacteal feeding, initiated breastfeeding later than the recommended guidelines [17,23,24]. The early breastfeeding initiation may actually help reduce pre-lacteal feeding. The breastfeeding practices in India have shown considerable improvement, especially after the launch of the National Rural Health Mission in 2005. However, the recent data shows a stagnation in the levels of early breastfeeding initiation. At the same time, the pace of reduction in the pre-lacteal feeding too has slowed down.

The study found that the children delivered in the public health facilities have a higher chance of getting breastfeeding within one hour after birth. In contrast, the practice is less common for children born in private health facilities and is lowest for children not delivered in a health facility. Earlier studies have also found place of birth significantly associated with the timely initiation of breastfeeding [25,26]. Further, pre-lacteal feeding is less common among children born in public health facilities and is relatively at higher levels for children born in private health facilities. This suggests that the service providers in public health facilities follow the breastfeeding guidelines more strictly than those in private health facilities. The levels of timely initiation of breastfeeding were undesirably low, and the practice of giving pre-lacteal feeding existed even in tertiary-care hospitals [17].

The factors associated with timely initiation were higher maternal education, antenatal counselling, absence of obstetric problems, and vaginal delivery. In contrast, those associated with pre-lacteal feeding were lower maternal education and caesarean delivery [17,27–29]. A study found that breastfeeding initiation was delayed after birth because the mothers perceived colostrum to be harmful for child’s health and that the mother’s milk is ‘not ready’ until two to three days postpartum [30].

The prevalence of pre-lacteal is significantly high, and early initiation is low in C-section deliveries. Use of general or spinal anaesthesia for caesarean delivery and the trauma during surgery delay mothers’ recovery, which delays the early initiation of breastfeeding. Meanwhile, it is compensated by milk other than breastmilk [17,31,32]. Results show that the distribution of those items for which mothers’ breastmilk in critical conditions can compensate is more predominant, such as milk other than breastmilk and infant formula in C-section delivery. But in vaginal deliveries, other factors related to cultures such as honey and *janam ghutti* were more predominant. The study also notes a change in the distribution of the types of pre-lacteal feed over the study period. Earlier the items were more items related to cultural and traditional practices. With time, the distribution of honey, *janam ghutti*, sugar/glucose water is reduced, and milk other than breastmilk and infant formula has increased.

The role of health professionals during pregnancy and the first two days after delivery is visible in improving breastfeeding practices. The study found that mothers who received breastfeeding advice during pregnancy have elevated levels of early initiation of breastfeeding and lower levels of pre-lacteal feeding practice. The study revealed that the likelihood of breastfeeding initiation within one hour after birth was almost 21% higher and 19% lower for children when healthcare providers observed breastfeeding during the first two days after birth. There is a significant association between two important interventions of receiving prenatal counselling on breastfeeding and postnatal support to mothers, with early initiation of breastfeeding [25,33].

The study results show that early initiation of breastfeeding lowers neonatal mortality and thus provides compelling evidence supporting the critical role the timing of breastfeeding initiation, particularly within the first one hour can significantly reduce the risk of severe illnesses during the first month of life. Earlier studies also showed an increased risk of neonatal death by 33% when the breastfeeding initiation is delayed between 2 and 23 hours and doubles when the initiation is delayed beyond 24 hours [8,34,35]. This could be linked to the immune system which is still growing during infancy, making them highly vulnerable to diseases [36]. Breastmilk contains vital nutrients, antibodies, and other bioactive substances that work as a natural defence against illnesses and boost the immunological response during infancy [37]. Further, using the most recent available data of 2019-21, this study found that pre-lacteal feed does not influence child survival much. This could be due to the fact that the study found that the items given as pre-lacteal feed predominantly used in India are typically not harmful and can be compensated by mothers’ breastmilk.

We also acknowledge a few limitations for this study. First, this study considered any food given to the newborn within the first three days after birth other than breastmilk as pre-lacteal feed. This is mainly due to the fact that the survey collects information by asking ‘In the first three days after delivery, was the baby given anything to drink other than breast milk?’ Further, the survey does not provide information on timing. Second, the pre-lacteal and initiation of breastfeeding information is available only for the last birth in the NFHS data. Due to this, the estimated NMR is restricted only to the last birth. Third, the NFHS-3 provides only state level data. So, we could not provide district level analysis.

# **Conclusions**

The present study found that promoting early initiation of breastfeeding reduces the pre-lacteal feeding practices suggesting that a stringent focus on enhancing levels of early initiation of breastfeeding may yield reductions in levels of pre-lacteal feeding among the newborns. The study findings further indicate that the breastfeeding advice during pregnancy and breastfeeding observation by the health service provider after the birth significantly improve breastfeeding and pre-lacteal feeding practices. This supports that increased focus on these practices by the grassroot level heath workers at the time of visits can help accelerate reductions in neonatal mortality. However, there is a gap in the program implementation as it lacks universal adherence, more specifically in the private health facilities. Thus, it is of utmost importance that the future programs make concerted efforts and pay greater attention to ensure that the expectant mothers are duly advised on early initiation of breastfeeding and that the service providers observe the early initiation to ensure adherence. For example, it may be helpful to create the awareness and greater commitments among the service provides about the importance of early initiation and its adherence and how this can bring about positive changes in the health outcomes among newborns including reduced mortality. Additionally, supporting new mothers with proper guidance and resources may facilitate establishing successful breastfeeding routines.

The study also documents evidence on association between neonatal mortality and breastfeeding and pre-lacteal feeding practices in India. The neonatal mortality rate is found to be lower among newborn who were given breastmilk within one hour after birth. The study further noted, that the pre-lacteal feed did not influence neonatal survival, but early initiation of breastfeeding does. Although, pre-lacteal feeding practices may continue given cultural context of the practices, it may be essential to create awareness on the choice of pre-lacteal feeds that are harmless. It is suggested that there is a need to strengthen the intervention of early breastfeeding initiation through breastfeeding adviser and cohesive observance of the early initiation to reduce neonatal mortality.

**Acknowledgment**

None

**Reference**

1.	Perin J, Mulick A, Yeung D, Villavicencio F, Lopez G, Strong KL, et al. Global, regional, and national causes of under-5 mortality in 2000–19: an updated systematic analysis with implications for the Sustainable Development Goals. Lancet Child Adolesc Heal. 2022;6: 106–115. doi:10.1016/S2352-4642(21)00311-4

2.	United Nations Inter-agency Group for Child Mortality Estimation (UNIGME). ‘Levels & Trends in Child Mortality: Report 2021, Estimates developed by the United Nations Inter-agency Group for Child Mortality Estimation. New York; 2021.

3.	World Health Organization. Newborn Mortality. 2022. Available: https://www.who.int/news-room/fact-sheets/detail/levels-and-trends-in-child-mortality-report-2021

4.	Office of the Registrar General & Census Commissioner. SAMPLE REGISTRATION SYSTEM (SRS)-STATISTICAL REPORT 2020. India (ORGI); 2022.

5.	Million Death Study Collaborators. Causes of neonatal and child mortality in India: A nationally representative mortality survey. Lancet. 2010;376: 1853–1860. doi:10.1016/S0140-6736(10)61461-4

6.	Edmond KM, Kirkwood BR, Amenga-Etego S, Owusu-Agyei S, Hurt LS. Effect of early infant feeding practices on infection-specific neonatal mortality: an investigation of the causal links with observational data from rural Ghana. Am J Clin Nutr. 2007;86: 1126–1131. doi:10.1093/ajcn/86.4.1126

7.	Borg B, Gribble K, Courtney‐Haag K, Parajuli KR, Mihrshahi S. Association between early initiation of breastfeeding and reduced risk of respiratory infection: Implications for nonseparation of infant and mother in the COVID‐19 context. Matern Child Nutr. 2022;18. doi:10.1111/mcn.13328

8.	Debes AK, Kohli A, Walker N, Edmond K, Mullany LC. Time to initiation of breastfeeding and neonatal mortality and morbidity: a systematic review. BMC Public Health. 2013;13: S19. doi:10.1186/1471-2458-13-S3-S19

9.	Liu J, Leung P, Yang A. Breastfeeding and Active Bonding Protects against Children’s Internalizing Behavior Problems. Nutrients. 2013;6: 76–89. doi:10.3390/nu6010076

10.	Else-Quest NM, Hyde JS, Clark R. Breastfeeding, Bonding, and the Mother-Infant Relationship. Merrill Palmer Q. 2003;49: 495–517. doi:10.1353/mpq.2003.0020

11.	Thapa BR. Health factors in colostrum. Indian J Pediatr. 2005;72: 579–581. doi:doi.org/10.1007/BF02724182

12.	Uruakpa FO, Ismond MAH, Akobundu ENT. Colostrum and its benefits: a review. 2002. Available: www.elsevier.com/locate/nutres

13.	Edmond KM, Zandoh C, Quigley MA, Amenga-Etego S, Owusu-Agyei S, Kirkwood BR. Delayed breastfeeding initiation increases risk of neonatal mortality. Pediatrics. 2006;117. doi:10.1542/peds.2005-1496

14.	Sarkar D, Dalai C, Sarkar K, Das S, Banerjee S. Breastfeeding practices and infant feeding pattern of a tribal population region of eastern India. J Fam Med Prim Care. 2020;9: 4570. doi:10.4103/jfmpc.jfmpc_631_20

15.	Venugopal S, Arulparithi CS. A study on knowledge of breast feeding and complementary feeding practices among mothers in a rural area, Tamil Nadu, India. Int J Contemp Pediatr. 2018;5: 1790. doi:10.18203/2349-3291.ijcp20183395

16.	Garje R, Thitame S, Somasundaram K, Gautam L, Gite M. Infant feeding beliefs and practices among tribal mothers in Nashik district, Maharashtra. Int J Med Sci Public Heal. 2016;5: 2203. doi:10.5455/ijmsph.2016.10122015443

17.	Patel A, Banerjee A, Kaletwad A. Factors associated with prelacteal feeding and timely initiation of breastfeeding in hospital-delivered infants in India. J Hum Lact. 2013;29: 572–578. doi:10.1177/0890334412474718

18.	International Institute for Population Sciences (IIPS) and ICF. National Family Health Survey NFHS-5, 2019-21. Mumbai, India; 2021.

19.	Edmond KM, Kirkwood BR, Tawiah CA, Agyei SO. Impact of early infant feeding practices on mortality in low birth weight infants from rural Ghana. J Perinatol. 2008;28: 438–444. doi:10.1038/jp.2008.19

20.	Phukan D, Ranjan M, Dwivedi LK. Impact of timing of breastfeeding initiation on neonatal mortality in India. Int Breastfeed J. 2018;13: 27. doi:10.1186/s13006-018-0162-0

21.	StataCorp. Stata Statistical Software: Release 14. TX: StataCorp LP; 2015.

22.	ESRI. ArcGIS Desktop: Release 10. Environmenntal System Research Institute; 2011.

23.	Parashar A, Sharma D, Gupta A, Dhadwal S. Pre-lacteal feeding practices and associated factors in Himachal Pradesh. Int J Heal Allied Sci. 2017;6: 30–34. doi:10.4103/2278-344X.200205

24.	Abdel-Rahman ME, El-Heneidy A, Benova L, Oakley L. Early feeding practices and associated factors in Sudan: a cross-sectional analysis from multiple Indicator cluster survey. Int Breastfeed J. 2020;15: 41. doi:10.1186/s13006-020-00288-7

25.	Gupta A, Dadhich JP, Manazir Ali S, Thakur N. Skilled Counseling in Enhancing Early and Exclusive Breastfeeding Rates: An Experimental Study in an Urban Population in India. Indian Pediatr. 2019;56: 114–118. doi:10.1007/s13312-019-1482-x

26.	Patel A, Badhoniya N, Khadse S, Senarath U, Agho KE, Dibley MJ. Infant and Young Child Feeding Indicators and Determinants of Poor Feeding Practices in India: Secondary Data Analysis of National Family Health Survey 2005–06. Food Nutr Bull. 2010;31: 314–333. doi:10.1177/156482651003100221

27.	Patel A, Bucher S, Pusdekar Y, Esamai F, Krebs NF, Goudar SS, et al. Rates and determinants of early initiation of breastfeeding and exclusive breast feeding at 42 days postnatal in six low and middle-income countries: A prospective cohort study. Reprod Health. 2015;12: S10. doi:10.1186/1742-4755-12-S2-S10

28.	Devasenapathy N, Neogi SB, Soundararajan S, Ahmad D, Hazra A, Ahmad J, et al. Association of antenatal care and place of delivery with newborn care practices: evidence from a cross-sectional survey in rural Uttar Pradesh, India. J Heal Popul Nutr. 2017;36: 30. doi:10.1186/s41043-017-0107-z

29.	Senanayake P, O’Connor E, Ogbo FA. National and rural-urban prevalence and determinants of early initiation of breastfeeding in India. BMC Public Health. 2019;19: 896. doi:10.1186/s12889-019-7246-7

30.	Bandyopadhyay M. Impact of ritual pollution on lactation and breastfeeding practices in rural West Bengal, India. Int Breastfeed J. 2009;4: 2. doi:10.1186/1746-4358-4-2

31.	Nguyen PH, Keithly SC, Nguyen NT, Nguyen TT, Tran LM, Hajeebhoy N. Prelacteal feeding practices in Vietnam: challenges and associated factors. BMC Public Health. 2013;13: 932. doi:10.1186/1471-2458-13-932

32.	Hoang Nguyen PT, Binns CW, Vo Van Ha A, Nguyen CL, Khac Chu T, Duong D Van, et al. Caesarean delivery associated with adverse breastfeeding practices: a prospective cohort study. J Obstet Gynaecol (Lahore). 2020;40: 644–648. doi:10.1080/01443615.2019.1647519

33.	Namasivayam V, Dehury B, Prakash R, Becker M, Avery L, Sankaran D, et al. Association of prenatal counselling and immediate postnatal support with early initiation of breastfeeding in Uttar Pradesh, India. Int Breastfeed J. 2021;16: 26. doi:10.1186/s13006-021-00372-6

34.	Smith ER, Hurt L, Chowdhury R, Sinha B, Fawzi W, Edmond KM. Delayed breastfeeding initiation and infant survival: A systematic review and meta-analysis. Simeoni U, editor. PLoS One. 2017;12: e0180722. doi:10.1371/journal.pone.0180722

35.	Timing of initiation, patterns of breastfeeding, and infant survival: prospective analysis of pooled data from three randomised trials. Lancet Glob Heal. 2016;4: e266–e275. doi:10.1016/S2214-109X(16)00040-1

36.	Semmes EC, Chen J-L, Goswami R, Burt TD, Permar SR, Fouda GG. Understanding Early-Life Adaptive Immunity to Guide Interventions for Pediatric Health. Front Immunol. 2021;11. doi:10.3389/fimmu.2020.595297

37.	Molès J-P, Tuaillon E, Kankasa C, Bedin A-S, Nagot N, Marchant A, et al. Breastmilk cell trafficking induces microchimerism-mediated immune system maturation in the infant. Pediatr Allergy Immunol. 2018;29: 133–143. doi:10.1111/pai.12841
