# Supplementary material for: First 72-hours after birth: Newborn feeding practices and neonatal mortality in India
Source: PLoS One. 2023 Oct 5;18(10):e0292353. doi: 10.1371/journal.pone.0292353 (PMC10553319; doi:10.1371/journal.pone.0292353)
Supplement: Attachment — Submitted filename: Response to Editor.docx [file pone.0292353.s005.docx]

**Thank you to the academic editor for your valuable comments and suggestions. We have responded to each query and have modify the manuscript accordingly.**

**Note: The line numbers mentioned against every response is based on the clean version of the revised manuscript.**

**Editor comment 1**: It would be better if the authors briefly provide information on the study sample added in the analyses from all the rounds of the NFHS, before discussing the statistical analysis. This becomes more important to add, as there is no section in this paper discussing the sample characteristics, usually mentioned in the Results section.

**Response 1**: Thank you for the suggestion. We have included a brief on the same in the method section under a sub head ‘Study sample’ in the revised manuscript [Please see line nos. 106-115].

**Editor comment 2:** Before it please add a few sentences to describe the nature of the outcome variable, for which the Tobit Regression model has been opted as an appropriate method of analysis. As both the outcome variables seem to be typical dichotomous variables, not censored continuous variables. If it is not so, please briefly describe the rationale for selecting the Tobit regression model.

**Response 2:** Thank you for your suggestion. We have explained the same in the revised manuscript. We have now used logistic regression analysis to identify predictors of the early breastfeeding initiation and pre-lacteal feed and modified the text accordingly. [Please see line nos. 147-148 and Table 3]

**Editor comment 3**: Comparison of or change in the prevalence must be discussed in the same unit for a better understanding of the trend. The authors can show the change while comparing the district prevalence in 2015-16 with the district prevalence in 2019-21. Comparing state prevalence with district prevalence is not useful, although they can be mentioned in the text.

Please refer to this comment for the interpretation in the paragraph below too.

**Response 3:** Thank you for the suggestion. We have now modified figure 2 keeping NFHS4 and 5 results. [Please see figure 2]

**Editor comment 4**: Results must be interpreted in terms of the unit of the study sample. In this study, last-born children/newborns are the study sample unit, so these proportions should refer to the children, not their mothers, despite their numbers being the same.

**Response 4**: Thank you for pointing out. We have written the proportion referring the children/newborn babies throughout the text at all appropriate places in the revised manuscript [Please also see line nos. 197, 198, 213, 246, 250, 252-253].

**Editor comment** **5**: Please refer to the earlier comments. Results must be written considering the study unit - proportion here refers to children, not the mothers. This must be followed throughout the manuscript.

**Response 5**: Thanks. The same has been addressed. [Please also see response 4].

**Editor comment** **6**: Received more or less?

**Response 6:** Thank you for pointing out the missing word error. We have written “received breastmilk early” in the revised manuscript. [Please see line no 208]

**Editor comment 7**: This doesn't seem to be a correct interpretation of the regression coefficient; these are not the odds ratios.

Please refer to this comment for the following texts wherever it is applicable in the manuscript.

**Response 7:** Thank you for pointing out the mistake. We have corrected the interpretation of regression coefficient in the revised manuscript at all places. [Please see line nos. 291-304, 306-317]

**Editor comment 8**: The study sample, i.e., children, constitutes all those born during the five years preceding the respective NFHS. They are not those who were born during the survey period.

**Response 8:** Thank you for pointing out. We have modified the line. [Please see line nos. 285-287, 315-317]

**Editor comment 9**: Additionally, authors may also try showing the association between NMR and the coverage of early breastfeeding/pre-lacteal feeding using scatterplots and respective linear regression equations. State-level NMR on y-axis and state-level percentage of early breastfeeding/pre-lacteal feeding on x-axis can be illustrated using scatterplots.

**Response 9**: Thank you for your suggestion. We have now added Figure 3 that gives scatterplots showing the association between NMR and the coverage of early breastfeeding/pre-lacteal feeding. State-level NMR are on y-axis and state-level percentage of early breastfeeding/pre-lacteal feeding on x-axis. [Please see Figure 3]

**Editor comment 10**: This would be good to provide 95% CIs for the NMRs shown in Table 4.

**Response 10:** Thank you for your suggestion. We have provided 95% CIs for the NMRs shown in Table 4. [Please see Table 4]

**Editor comment 11**: Authors may thank two anonymous reviewers for their suggestions.

**Response 11**: Thank you for your suggestion. We have acknowledged two anonymous reviewers and academic editor for their suggestions. [Please see line nos. 449-450]
